# Congo red test for identification of preeclampsia: Results of a prospective diagnostic case-control study in Bangladesh and Mexico

**DOI:** 10.1016/j.eclinm.2020.100678

**Published:** 2020-12-22

**Authors:** Hillary Bracken, Irina A. Buhimschi, Anisur Rahman, Patricio R. Sanhueza Smith, Jesmin Pervin, Salma Rouf, Manuel Bousieguez, Lourdes García López, Catalin S. Buhimschi, Thomas Easterling, Beverly Winikoff

**Affiliations:** a Gynuity Health Projects, 220 East 42nd Street, Suite #710, New York, NY 10017, USA; b Department of Obstetrics and Gynecology, University of Illinois at Chicago, College of Medicine, Chicago, IL 60612, USA; c Maternal and Child Health Division, icddr,b, Dhaka, Bangladesh; d Mnistry of Health, Mexico City, Altadena 23, Nápoles, 03810 Ciudad de México, CDMX; e Department of Obstetrics and Gynecology, Dhaka Medical College and Hospital, Dhaka, Bangladesh; f Hospital Materno-Infantil Inguarán, Ministry of Health, Mexico City, Mexico; g University of Washington, Seattle, WA 98195, USA

## Abstract

**Background:**

Misfolded proteins in the urine of women with preeclampsia bind to Congo Red dye (urine congophilia). We evaluated a beta prototype of a point-of-care test for the identification of urine congophilia in preeclamptic women.

**Methods:**

Prospective diagnostic case-control study conducted in 409 pregnant women (*n* = 204 preeclampsia; *n* = 205 uncomplicated pregnancies) presenting for delivery in two tertiary level hospitals located in Bangladesh and Mexico. The GV-005, a beta prototype of a point-of-care test for detecting congophilia, was performed on fresh and refrigerated urine samples. The primary outcome was the prevalence of urine congophilia in each of the two groups. Secondary outcome was the likelihood of the GV-005 (index test) to confirm and rule-out preeclampsia based on an adjudicated diagnosis (reference standard).

**Findings:**

The GV-005 was positive in 85% of clinical cases (83/98) and negative in 81% of clinical controls (79/98) in the Bangladesh cohort. In the Mexico cohort, the GV-005 test was positive in 48% of clinical cases (51/106) and negative in 77% of clinical controls (82/107). Adjudication confirmed preeclampsia in 92% of Bangladesh clinical cases (90/98) and 61% of Mexico clinical cases (65/106). The odds ratio of a urine congophilia in adjudicated cases versus controls in the Bangladesh cohort was 34^.^5 (14^.^7 – 81^.^1) (*p*<0^.^001) compared to 4^.^2 (2^.^1 – 8^.^4; *p*<0^.^001) in the Mexico cohort.

**Interpretation:**

The GV-005, a beta prototype of a point-of-care test for detection of urine congophilia, is a promising tool for rapid identification of preeclampsia.

**Funding:**

Saving Lives at Birth.

**Research in context**

**Evidence before this study**

Preeclampsia is a pregnancy-specific hypertensive disorder and a leading cause of maternal and perinatal morbidity and mortality worldwide. Several inflammatory and placental biomarkers have been identified as potentially useful tools for the prediction or diagnosis of preeclampsia. Previous research showing that misfolded proteins in the urine of women with preeclampsia bind to Congo Red dye has been applied to develop laboratory-based techniques and a paperbased test for use at the bedside.

**Added value of this study**

This study reports the diagnostic characteristics of a prototype of a point-of-care test for detection of urine congophilia when used in low and middle income country setting populations. The findings suggest urine congophilia can be rapidly identified using a beta prototype of a lateral flow diagnostic device, GV-005.

**Implications of all available evidence**

Failure to diagnosis, misdiagnosis, delay in transfer, and receipt of unnecessary treatment contribute to high rates of maternal and neonatal morbidity and mortality associated with preeclampsia. Our study shows that a point-of-care diagnostic for detection of urine congophilia has the potential to improve the triage and diagnosis of patients with preeclampsia. A point-of-care test for the detection of urine congophilia could aid in the clinical care of preeclamptic women and reduce the morbidity and mortality associated with this disease.

Alt-text: Unlabelled box

## Introduction

Preeclampsia is a pregnancy-specific hypertensive disorder and a leading cause of maternal and perinatal morbidity and death worldwide: The World Health Organization (WHO) estimates that 16% of global maternal mortality (~ 63,000 maternal deaths annually) is due to preeclampsia [1]. Traditional diagnoses have relied upon clinical characteristics such as hypertension and proteinuria. However, urine dipsticks and evaluation of spot protein-to-creatinine (P:C ratio) are known to associate with false positive and false negative results [2, 3, 4]. In high resource settings, ambiguity in diagnosis of preeclampsia generally leads to increased use of health-care resources with hospital admission for antenatal monitoring and increased early delivery for preeclampsia even when patients present with preeclampsia imitators [5]. In low-resource settings, diagnostic uncertainty may delay the receipt of appropriate care, contributing to near-miss events and high mortality [6].

Several inflammatory and placental biomarkers have been identified as potential tools for prediction or diagnosis of preeclampsia [7]. Commercially available tests for these biomarkers are laboratory-based, require a blood sample or are employed as part of complex algorithms making them impractical as point-of-care tests, especially for low-resource settings. A new diagnostic technology that circumvents these limitations may offer significant advantages if able to maintain the dipstick's ease of use. Women with preeclampsia excrete high amounts of misfolded protein in urine, as a consequence of increased protein misfolding load, a phenomenon more similar to Alzheimer's or prion disease [8,9]. These misfolded proteins have a propensity to bind to Congo Red (CR) dye (congophilia) [9]. Based on this principle, our team developed and tested a urine paper-based point-of-care test for rapid screening and identification of preeclampsia, independent of clinical criteria [10,11]. The presence of congophilia among patients diagnosed with preeclampsia has been further validated by other groups in research laboratory settings using our initially reported nitrocellulose-based procedure [12, 13, 14] or a different device intended for point-of-care [15].

We hypothesized that preeclampsia is characterized by urine congophilia which can be rapidly identified using a beta prototype of a lateral flow diagnostic device, GV-005 (GestVision Inc, Groton, CT), for improved diagnosis of preeclampsia. The study objectives were: 1) to determine the prevalence of urine congophilia in tertiary facility settings in two low-resource countries (Bangladesh and Mexico) and 2) to compare the Congo Red test results to a clinical diagnosis of preeclampsia.

## Methods

### Study design and participants

We conducted a prospective diagnostic case-control study in the Labor and Delivery units at Dhaka Medical College in Dhaka, Bangladesh (April-July 2017) and the Hospital Materno Infantil Inguarán, Mexico City, Mexico from (July 2016 - September 2018). Both public hospitals serve as tertiary referral centers. Data collection was planned before the index test, GV-005, was performed. At both study sites, research assistants identified preeclampsia cases (with or without severe features) based on the facility's standard diagnostic criteria. For each preeclampsia case, a control patient was enrolled on the same day from women admitted for labor induction or elective caesarean section of a normal term baby. Women who agreed to provide a urine sample antepartum and were of the age eligible to consent were asked to participate. All participants provided individual written consent. The trial was approved by the Research and Ethics Committee at Dhaka Medical College and the Institutional Review Board of Mexico City's Secretariat of Health. This trial was registered as clinicaltrials.gov NCT02381210.

### Procedures

**Fig. 1:**
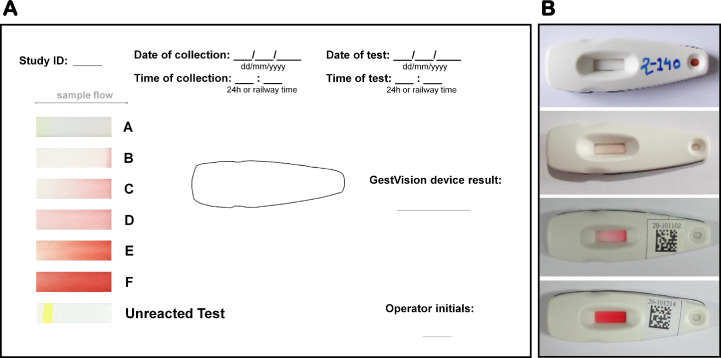
Visual chromatic aid for interpretation of GV-005 and sample test devices.

Research assistants approached eligible women immediately after confirmation of preeclampsia diagnosis or admission for delivery in the case of normal controls. After signing the consent form, the research assistant asked women to provide a urine (~10 mL) sample in a sterile 15 mL container. The research assistant labeled the sample with a unique study ID number and then transported it to a research laboratory within the same facility for immediate processing or refrigeration (contingent on time of collection and staff availability). Within 72-hours from urine collection, a laboratory technician (different than the research assistant) tested for congophilia using a research beta prototype lateral flow diagnostic device (GV-005, GestVision, USA) [16]. All laboratory staff was certified on the use of the device and interpretation of the result prior to trial initiation. The laboratory technician applied 100 microliters of urine to the test casette using the test dropper, read the device at 3 min and scored its result on a six point scale (A-F) using a visual chromatic aid on the result recording sheet (Fig. 1). The technical performance of the GV-005 was investigated in a prior pilot study vis-à-vis the in-house paper test kit [9] and the research laboratory nitrocellulose procedure [17]. Additional information about this and the results obtained appear in Supplement A. Based on this, we considered scores A or B negative for congophilia and scores of C-F positive for congophilia. Each sample was tested only once unless there was a technical issue (persistence of the blue control line) or the result was indeterminate. In this case the same sample was retested using a new device. Research laboratory staff was blinded to the clinical course of participating women and the clinicians and the research assistant were blinded to the GV-005 test results. The diagnosis of preeclampsia that resulted in the enrolment of cases and controls was left to the judgement of local providers and based on the standard diagnostic criteria at each facility. A standard proteinuria test was performed at multiple points during patient care, including at the time of diagnosis and at the time of urine collection for this trial. Similarly, clinicians undertook treatment decisions as per the local standard of care and all medical decisions were taken independent of the study protocol.

Research staff collected data on patient history, preeclampsia diagnosis, and labor and delivery outcomes transcribed from patient charts using standardized paper data collection forms. Incorrect classification of outcomes can result in the biased performance of a diagnostic test or reduced power [18]. Thus, each case and control was independently adjudicated by two obstetricians one of whom was a certified Maternal Fetal Medicine specialist. Similar approaches were employed at both sites. Both adjudicators were blinded to the congophilia test result and the enrolling provider's diagnosis. The adjudicators reviewed data collected on the trial's standard data collection forms and classified cases and controls using the American College of Obstetricians and Gynecologists (ACOG) Task Force definition of hypertensive disorders of pregnancy [19]. Details are provided in Figs. S1 and S2.

### Main outcome measure

The primary outcome was the prevalence of urine congophilia in each of the two groups. Secondary outcome was the likelihood of the GV-005 (index test) to confirm and rule-out preeclampsia based on the adjudicated diagnosis (reference standard).

### Statistical analysis

We based our sample size on our prior experience with identification of bio-markers specific for preeclampsia [9]. In the prior study, the Congo Red Dot test resulted in an AUC of 0^.^894 (95%CI: 0^.^866–0^.^918) compared to the standard proteinuria dipstick (0^.^825 (0^.^789–0^.^856)) (p-value<0^.^05) [9]. Based on these results, we projected that the study required 165 women in each arm to see a significant difference in the predictive value of the Congo Red Dot test (power: 0^.^8 and alpha of 5%).   Additional patients were recruited to strengthen the conclusions of the analysis. Differences between the two groups were compared using chi-square (or Fischer's exact) and Student *t*-test for categorical and continuous variables, respectively using SPSS Statistics 20 (SPSS Inc. Chicago, IL) statistical software. Odds ratios or the likelihood of the GV-005 (index test) to confirm and rule-out preeclampsia based on an adjudicated diagnosis (reference standard) were calculated using MedCalc statistical software.

### Role of the funding source

The funder of the study had no role in the study design, data collection or analysis. The corresponding author had full access to all the data in the study and had final responsibility for the decision to submit for publication.

## Results

**Fig. 2:**
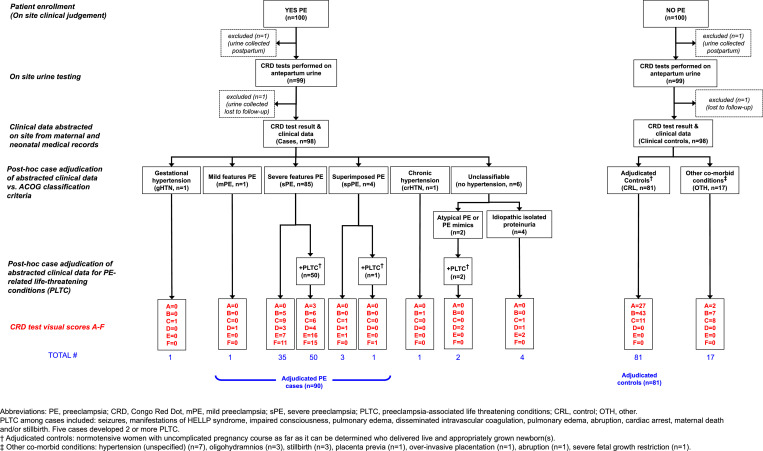
Study flowchart: Bangladesh.

**Fig. 3:**
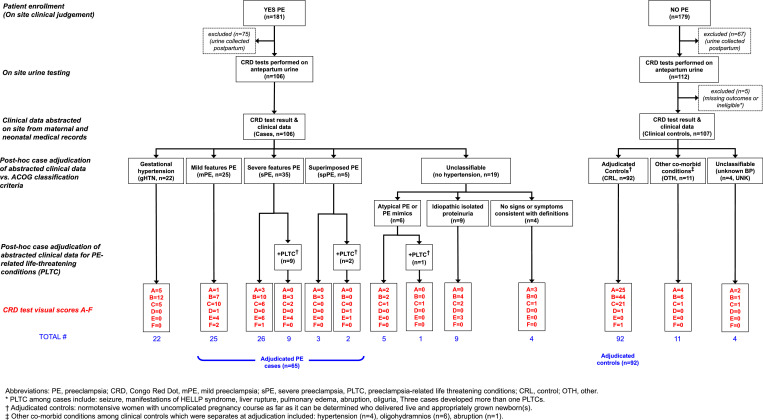
Study flowchart: Mexico.

Between July 11 2016, and Sept 25 2018, 560 women in two hospitals were enrolled in the study (Fig. 2, Fig. 3). 144 women were excluded because the urine sample was collected postpartum. There were no missing data of the index test. Five patients recruited as controls at the Mexico site were missing clinical data necessary for adjudicators to either rule out or confirm preeclampsia and thus excluded. Two women recruited in Bangladesh were excluded because they were lost to follow-up. In Bangladesh, 64.3% of cases (63/98) and 53.1% of controls (52/98) were tested after refrigeration (*p* = 0.111). In Mexico, 81% of cases (85/105) and 66.4% of controls were tested after refrigeration (71/107) (*p* = 0.012). Although there is no systematic data on the effect of refrigeration on CR results using the next generation GV-005 device, we do not expect the short term refrigeration to affect the results. One case from Mexico was missing data on whether the sample was tested after refrigeration. In five cases, the result of the GV-005 test was indeterminate (i.e. showed a persistent blue control line) and the laboratory technician repeated the test with a new device using the same urine sample and results included in the analysis. Clinical data was abstracted on site from maternal and neonatal medical records for 409 women. A flowchart of the study population is presented in Fig. 2 (Bangladesh) and 3 (Mexico).

### Patient characteristics

Demographic and clinical characteristics of the patients enrolled in the study with complete clinical data are presented in Table 1 (Bangladesh) and Table 2 (Mexico). The Bangladesh cohort included 98 preeclampsia cases and 98 controls based on the clinical diagnosis. In Bangladesh, 55% of cases and 39% of controls were nulliparous. Approximately half (51%) of cases recruited in Bangladesh were less than 34 weeks gestation at the time of the urine sample collection. Most controls (64%) in the Bangladesh cohort were recruited at term. Among preeclampsia cases in the Bangladesh cohort, the most common signs and symptoms at the time of diagnosis were elevated blood pressure (98% or 96/98), proteinuria (96% or 94/98), headache (76% or 74/98) and changes in vision (66% or 65/98) or seizure (40% or 39/98). Most preeclampsia cases (92%) had received magnesium sulfate prior to study recruitment and urine sample collection. Over half (54%) of Bangladesh cases experienced a preeclampsia-related life threatening condition (PLTC) including seizure (44%), hemolysis, elevated liver enzymes and low platelet count (HELLP) syndrome (7%), impaired consciousness (1%), pulmonary edema (2%), disseminated intravascular coagulation (1%), abruption (1%), cardiac arrest (1%), maternal death (1%) and/or stillbirth (45%). Five cases developed 2 or more conditions. The rate of pregnancy-related complications among the control group was low and included one case of hypovolemic shock (who also underwent a hysterectomy) (1%), uterine hyperstimulation (1%), and placental abruption (1%). Most controls in the Bangladesh cohort had a live birth (97%) and all babies were alive at the time of discharge.

**Table 1:** Characteristics of women enrolled in the case control study in Bangladesh. (n,%).

Variable	Case (*n* = 98)	Control (*n* = 98)	p-value
Maternal age (years) (SD) (range)	25·3 (5^.^2)(17–35)	25^.^5(4^.^8)(18–38)	0^.^754
Gestational age (completed weeks)			
<34 weeks 34–36 weeks 37–38 weeks 39≥ weeks	50 (51·0) 32 (32·7) 6 (6·1) 10 (10·2)	0 (0) 0 (0) 35 (35·7) 63 (64·3)	<0·001
Multiple gestation	5 (10^.^4)	2 (3^.^6)	0·244
Parity (n,%)			
0 1 2 3+	55 (56^.^1) 23 (23·5) 15 (15·3) 5 (5·1)	39 (39·8) 29 (29·6) 27 (27·6) 3 (3·1)	0·062
Pre-existing conditions before pregnancy			
diabetes chronic hypertension renal disease liver disease other*	0 5 (5·1) 0 0 2 (2·0)	0 2 (2·0) 0 0 4 (4·0)	- 0.248 - - 0.407
Preeclampsia diagnosis by enrolling provider			
Mild preeclampsia Severe preeclampsia HELLP Eclampsia Preeclampsia uncategorized	1 (1·0) 22 (22·4) 8 (8·2) 39 (39·8) 28 (28·6)	- - - - -	- - - - -
Signs and symptoms at time of diagnosis			
Elevated BP Proteinuria Headache Changes in vision Abdominal pain Nausea/vomiting Seizure	96 (98·0) 94 (95·9) 74 (75·5) 65 (66·3) 1 (1·0) 29 (29·6) 39 (39·8)	- - - - - - -	- - - - - - -
Systolic BP (mmHg) at time of diagnosis			
≤139 140–159 ≥160	8 (8·2) 33 (33·7) 57 (58·2)	- - -	- - -
Diastolic BP (mmHg) at time of diagnosis			
≤89 90–109 ≥110	10 (10·2) 36 (36·7) 52 (53·1)	- - -	- - -
Proteinuria at time of diagnosis			
Nil or Trace +1 +2 ≥+3	10 (10·2) 10 (10·2) 8 (8·2) 70 (71·4)	- - - -	- - - -
Received MgSO4 ≤6 h preceding enrollment	90 (91·8)	–	–
Received antihypertensive drug ≤6 h hours preceding enrollment	98 (100·0)	–	–
Mode of delivery			0·115
Vaginal delivery Forceps or vacuum delivery C-section	48 (49·0) 0 50 (51·0)	35 (35·7) 1 (1·0) 62 (63·3)	
Complications of labor and delivery			
Uterine hyperstimulation FHR abnormality Uterine rupture Placental abruption Diagnosis of PPH (>500 ml) Manual removal of placenta Blood products (blood plasma, platelets or red blood cells) Severe hypertension (single systolic BP≥160 or diastolic BP≥110) after enrolment Hypovolemic shock	0 0 0 1 (1·0) 1 (1·0) 0 34 (34·3) 0 0	1 (1·0) 5 (5·1) 0 1 (1·0) 0 0 14 (14·3) 0 1 (1·0)	0·316 0·024 - 1·00 0·316 - 0·001 - 0·316
Seizure after admission to labor and delivery	4 (4·0)	0	0.043
HELLP syndrome	7 (7·1)	0	0·007
Pulmonary edema	2 (2·0)	0	0·155
DIC based on clinical assessment	1 (1·0)	0	0·316
Received MgSO4 at time of delivery	48 (49·0)	1 (1·0)	<0·001
Received antihypertensive drugs at time of delivery	81 (81·6)	2 (2·0)	<0·001
Admission to ICU up to discharge	5 (5·1)	1 (1·0)	0·097
Indication for admission to ICU			
Uncontrolled hypertension HELLP Postpartum eclampsia, DIC Cardiac arrest Postpartum cardiomyopathy Total abdominal hysterectomy/hypovolemic shock	1 (1·0) 1 (1·0) 1 (1·0) 1 (1·0) 1 (1·0) 0	0 0 0 0 0 1 (1·0)	
Maternal death	1 (1·0)	0	0·316
Delivery outcome^a^ Live birth Stillborn	*n* = 103 58 (56·3) 45 (43·7)	*n* = 99 96 (96·9) 3 (3·1)	<0.001
Neonatal morbidity^a^			
Apgar<7 at 5 min Meconium-stained liquor (any grade) Neonatal convulsions Clinically diagnosed birth asphyxia Clinically diagnosed septicemia Other	1 (1·7) 2 (3·1) 1 (1·0) 3 (3·1) 0 1	0 0 0 0 0 3 (3·1)	
Baby admitted to special care nursery^a^	30 (30·6)	5 (5·2)	<0·001
Baby given oxygen^a^	24 (24·5)	4 (4·1)	<0·001
Neonatal deaths prior to discharge^a^	8 (8·1)	0	<0·001

Abbreviations**:** BP**=**Blood pressure, MgSO4=magnesium sulfate, FHR=fetal heart rate, PPH=postpartum hemorrhage (>500 ml blood loss), DIC=Disseminated intravascular coagulation,ICU=intensive care unit. Pulmonary edema defined as oxygen saturation <90% and an abnormal chest x-ray.

a Sample size of babies is 103 born to women in the case group and 99 babies born to women in the control group due to loss to follow-up (*n* = 1) and multiple pregnancies (case: 5; control: 2).

**Table 2:** Characteristics of women enrolled in case control study in Mexico(n,%).

Variable	Case (*n* = 106)	Control (*n* = 107)	p-value
Mean maternal age (years) (SD) (range)	25·0(6·2)(13–39)	23·6(6·2)(14–42)	0·123
Gestational age (completed weeks)			
<34 weeks 34–36 weeks 37–38 weeks 39≥ weeks	14 (13·2) 18 (17·0) 33 (31·1) 41 (38·7)	7 (6·5) 15 (14·0) 21 (19·6) 64 (59·8)	0·016
Multiple pregnancy (n,%)	1 (0·9)	0	0·187
Parity (n,%)			
0 1 2 3+	58 (54·7) 37 (34·9) 8 (7·5) 3 (2·8)	52 (48·6) 23 (21·5) 22 (20·6) 10 (9·3)	0.003
Pre-existing conditions before pregnancy			
diabetes chronic hypertension renal disease liver disease other	1 (0·9) 5 (4·7) 0 0 6 (5·7)	1 (0·9) 0 0 0 7 (6·5)	0·995 0·023 - - 0·788
Preeclampsia diagnosis at time of enrollment			
Mild preeclampsia Severe preeclampsia Eclampsia Preeclampsia uncategorized	21 (19·8) 70 (66·0) 2 (1·9) 13 (12·3)	- - - -	- - - -
Signs and symptoms at time of diagnosis			
Elevated BP Proteinuria Headache Changes in vision Abdominal pain Nausea/vomiting Seizure	88 (83·0) 6 (5·7) 23 (21·7) 2 (1·9) 11 (10·4) 2 (1·9) 1 (0·9)	- - - - - - -	- - - - - - -
Systolic BP (mmHg) at time of diagnosis			
≤139 140–159 ≥160	26 (24·5) 51 (48·1) 29 (27·4)	- - -	- - -
Diastolic BP (mmHg) at time of diagnosis			
≤89 90–109 ≥110	32 (30·2) 62 (58·5) 12 (11·3)	- - -	- - -
Proteinuria at time of diagnosis			
Nil or Trace +1 +2 >+3	26 (24·5) 43 (40·6) 14 (13·2) 22 (20·8)	- - - -	- - - -
Received MgSO4 ≤6 h preceding enrollment (n,%)	62 (58·5)	–	–
Received antihypertensive drug ≤6 h hours preceding enrollment	19 (17·9)	–	–
Mode of delivery			
Vaginal delivery Forceps or vacuum delivery C-section	23 (21·7) 2 (1·9) 81 (76·4)	64 (59·8) 5 (4·7) 38 (35·5)	*p*<0·001
Complications of labor and delivery			
Uterine hyperstimulation FHR abnormality Uterine rupture Placental abruption Diagnosis of PPH (>500 ml) Manual removal of placenta Blood products (blood plasma, platelets or red blood cells) Severe hypertension (single systolic BP≥160 or diastolic BP≥110) Other	0 0 0 1 (0·9) 10 (9·34) 0 4 (3·8) 4 (3·7) 6 (5·6)	2 (1·9) 0 1 (0·9) 1 (0·9) 3 (2·8) 0 0 0 4 (3·7)	0·157 - 0·318 0·995 0·043 - 0·043 0·043 0·507
Seizure up to discharge	2 (1·9)	0	0·153
Suspected cerebral edema or cerebral hemorrhage	0	0	–
HELLP syndrome	6 (5·7)	0	0·013
Pulmonary edema (O2 sat <90%, abnormal chest xray)	1 (0·9)	0	0·314
Oliguria (<25 cc/hr for 2 hr) up to 2 h after end of study period	2 (1·9)	0	0·153
DIC based on clinical assessment	0	0	–
Dialysis	0	0	–
Received MgSO4 at time of delivery	63 (59·4)	1 (0·9)	<0·001
Received antihypertensive drugs at time of delivery	86 (81·1)	1 (0·9)	<0·001
Admission to ICU up to discharge	32 (30·2)	2 (1·9)	<0·001
Indication for admission to ICU			
Uncontrolled hypertension PE/Eclampsia HELLP Other	5 (4·7) 18 (16·9) 4 (3·7) 4 (3·7)	0 1 (0·9) 0 1 (0·9)	
Maternal death	0	0	–
Outcome of delivery^a^			
Live birth Stillborn	106 (99·1) 1 (0·9)	107 (100) 0	0·314
Neonatal morbidity^a^			
Apgar<7 at 5 min Meconium-stained liquor (any grade) Neonatal convulsions Clinically diagnosed birth asphyxia Clinically diagnosed septicemia Other	3 (2·9) 10 (9·4) 0 4 (3·8) 0 9 (8·5)	0 9 (8·4) 0 2 (1·9) 0 4 (3·7)	
Baby admitted to special care nursery^a^	17 (16·0)	7 (6·5)	0·028
Baby given oxygen^a^	20 (18·9)	4 (3·7)	<0·001
Baby ventilated^a^	13 (12·3)	3 (2·8)	<0·001
Neonatal deaths prior to discharge^a^	4 (3·8)	1 (0·9)	0·171

Abbreviations**:** BP**=**Blood pressure, MgSO4=magnesium sulfate, FHR=fetal heart rate, PPH=postpartum hemorrhage (>500 ml blood loss), DIC=Disseminated intravascular coagulation,ICU=intensive care unit. Pulmonary edema defined as oxygen saturation <90% and an abnormal chest x-ray.

a Sample size of babies is 107 born to women in the case group and 107 babies born to women in the control group.

The Mexico cohort included 106 cases and 107 controls. Table 2 describes the demographic characteristics and delivery outcomes of the cases and controls recruited in Mexico. In the Mexico cohort, 53% of cases and 50% of controls were nulliparous. Over half of preeclampsia cases (70%) in the Mexico cohort were enrolled and delivered at 37 weeks gestation or more. The most common preeclampsia signs and symptom at the time of diagnosis among cases recruited in Mexico were elevated blood pressure (83%) or headache (22%). Slightly over half of the preeclampsia cases (57%) were treated with magnesium sulfate prior to enrolment. Approximately 11% of cases recruited in Mexico experienced a preeclampsia-related life-threatening condition including seizure (3%), HELLP syndrome (6%), liver rupture (1%), pulmonary edema (1%), abruption (1%), oliguria (2%) and stillbirth (1%). Three cases developed more than one condition. Among the Mexico control group, very few women developed pregnancy-related complications including uterine rupture (1%), placental abruption (1%) or postpartum hemorrhage (3%). All women with an uncomplicated pregnancy had a live birth at delivery; the rate of neonatal death was also low (0^.^9%).

Table S1 compares the profile of preeclampsia cases recruited at the two sites. A greater proportion of women with preeclampsia recruited at the study site in Bangladesh (40%) exhibited signs of advanced disease, i.e. seizure, compared to their Mexican counterparts (1%) (*p*<0^.^001). Although elevated blood pressure was listed as the most common criteria for diagnosis in both cohorts, the proportion of cases with severe hypertension (systolic >=160 mmHg or diastolic >=90 mmHg blood pressure) was significantly lower in Mexico (severe systolic blood pressure: 29% or severe diastolic blood pressure: 12%) compared to cases in Bangladesh (severe systolic blood pressure: 57% or severe diastolic blood pressure: 53%) (both *p*<0^.^001). Women with a clinical diagnosis of preeclampsia recruited in Mexico were also more likely to be diagnosed based on less specific symptoms such as abdominal pain (10%) compared to their counterparts in Bangladesh (1%) (*p* = 0.005). Many cases in Mexico (25%) were diagnosed with preeclampsia in the absence of proteinuria. Although a greater proportion of cases in Bangladesh experienced a preeclampsia-related life-threatening condition, intensive care unit admissions were significantly higher among preeclampsia cases in Mexico (30%) compared to Bangladesh (2%) (*p*<0.001).

### Patient profile and adjudicated diagnosis, and GV-005 test result

**Fig. 4:**
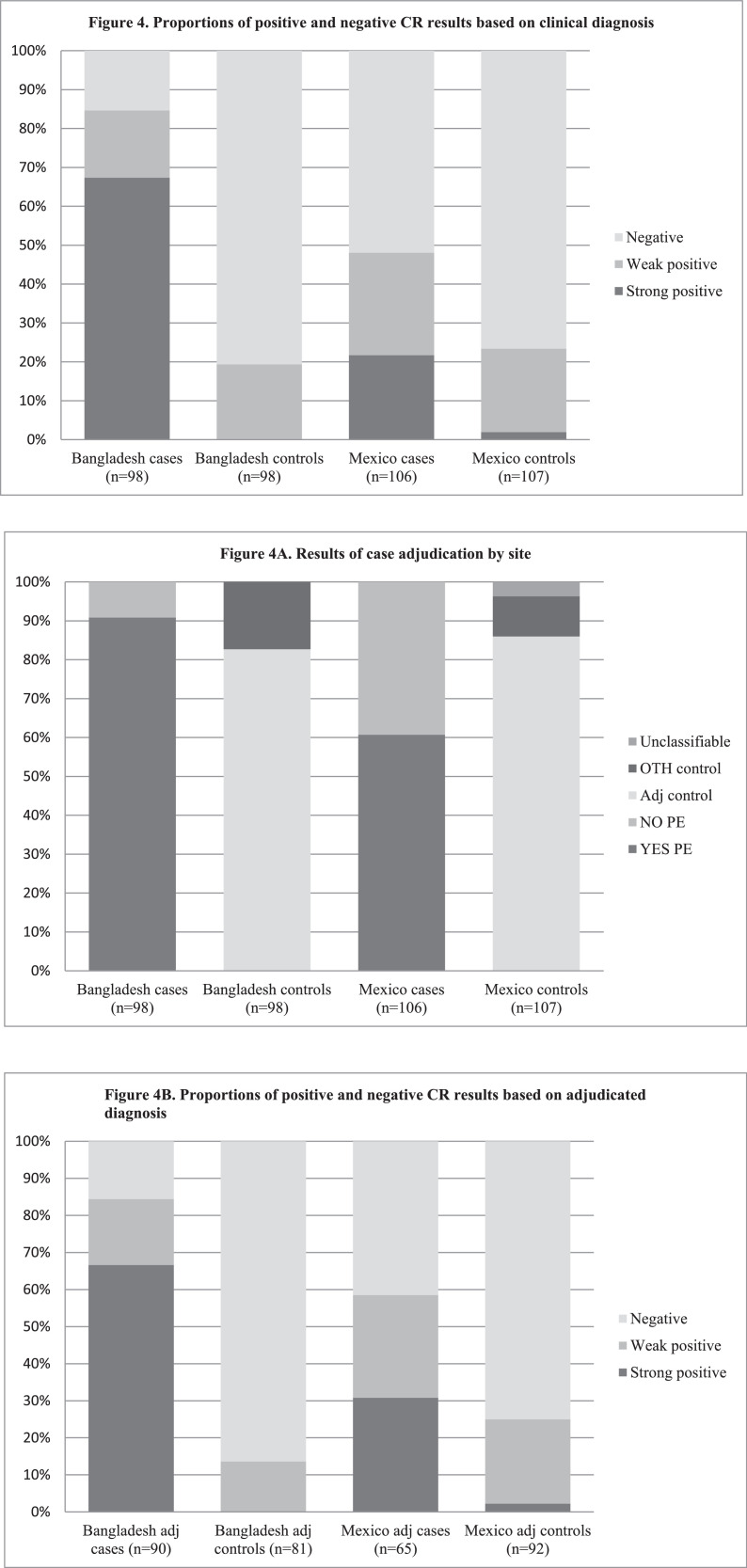
Proportions of positive and negative CR results based on clinical diagnosis . A. Results of case adjudication by site. B. Proportions of positive and negative CR results based on adjudicated diagnosis.

The breakdown of the population for comparison of GV-005 test results, based on the final adjudicated diagnosis, is presented in Fig. 2 (Bangladesh) and Fig. 3 (Mexico). Fig. 4 describes the proportion of cases and controls at each site with a positive test result. The prevalence of a urine congophilia (primary outcome) among cases in Bangladesh was 85% (83/98) and 48% (51/106) in Mexico. Conversely, urine congophilia was not present in 81% of controls (79/98) in Bangladesh and in 77% of controls (82/107) in Mexico.

Adjudication using the ACOG criteria confirmed preeclampsia in 92% of cases in Bangladesh (90/98) and 61% of cases in Mexico (65/106) (*p*<0.001) (secondary outcome). The diagnostic results of the CR test based on the final adjudicated diagnosis using the ACOG criteria are presented in Table 3. In Bangladesh, the odds ratio of urine congophilia in adjudicated cases versus controls was 34.5 to 1 (95% CI: 14.7–81.1) (secondary outcome). We conducted exploratory analyses and found that a better model for preeclampsia diagnosis can be achieved using the higher cut-off of D-F compared to C-F. With a higher cut-off, and only including results D-F as positive for congophilia, the odds of a positive result in adjudicated cases versus controls in the Bangladesh cohort was higher (323 to 1) (95% CI: 19.4–5393.1) (Table 3). In Mexico, the odds ratio of a positive result in adjudicated cases versus controls was 20 to1 (95% CI: 4^.^5–89^.^4) using the cut-off of D-F and 4.2 to 1 (95% CI: 2^.^1–8^.^0. when C-F was defined as a test positive for congophilia.

**Table 3:** Diagnostic characteristics of the Congo Red test by site of recruitment.

Test Score	Odds Ratio	Sensitivity (%)	Specificity (%)	+LR	-LR
	[95%CI]	[95%CI]	[95%CI]	[95%CI]	[95%CI]
***Bangladesh***					
D – F	323·3 *	66·7	100	‬‬∞	0·33
	[19·4 – 5393·1]	[56·9 – 76·4]	[100·0 – 100·0]		[0·3 – 0·4]
C – F	34·5 *	84·4	86·4	6·22	0·18
	[14·7 – 81·1]	[77·0 – 91·9]	[79·0 – 93·9]	[3·6 – 10·8]	[0·1 – 0·3]
***Mexico***					
D – F	20·0 *	30·8	97·8	14·2	0·7
	[4·48 – 89·4]	[19·6 – 42·0]	[95·8 – 1·0]	[3·4 – 58·5]	[0·6 – 0·8]
C – F	4·2 *	58·5	75·0	2·3	0·6
	[2·1 – 8·4]	[46·5 – 70·4]	[66·2 – 83·9]	[1·6 – 3·5]	[0·4 - 0·8]

The reference test for cases was the diagnosis of preeclampsia at post-hoc adjudication (mild, severe or superimposed) (Bangladesh, *n* = 90 and Mexico, *n* = 65). The absence of preeclampsia was considered only in adjudicated controls (Bangladesh, *n* = 81 and Mexico, *n* = 92). The GV-005 test was evaluated as the index test. * *p*<0.001.

Abbreviations: CI=confidence interval; +LR= positive likelihood ratio; -LR= negative likelihood ratio.

### Breakdown characteristics of cases, clinical diagnosis and adjudicated diagnosis by GV-005 test result

Table 4 presents the grouping of cases based on the attending team's diagnosis, final adjudicated diagnosis using ACOG criteria and result of the GV-005 test by site. In the Bangladesh control group 81 (83%) were adjudicated as non-preeclampsia and 69 of 81 (85%) adjudicated controls tested negative for congophilia. In the control group, a positive result was observed in 11 adjudicated controls (14%). Case by case analysis found that control patients who tested positive included patients with oligohydramnios, a history of hypertension and heart disease, and gestational hypertension. Amongst Bangladesh preeclampsia cases, 8 cases (8%) were adjudicated as non-preeclampsia. In this sub-group, 1 patient had a negative test result. The CR result concurred with the adjudicated diagnosis of chronic hypertension. In the group of cases adjudicated as non-preeclampsia but with a positive CR result, 7 patients tested weak positive for congophilia including patients diagnosed with gestational hypertension, atypical preeclampsia or preeclampsia mimics and idiopathic isolated proteinuria. Amongst preeclampsia cases where the enrolling diagnosis concurred with the adjudicated diagnosis (*n* = 90), 84% had a positive GV-005 test result. Adjudicated cases who tested negative for congophilia included cases of severe preeclampsia with and without PLTC (including one death due to cardiac arrest) and 5 cases with a history of eclampsia.

**Table 4:** Breakdown of cases by final adjudicated diagnosis and Congo Red test result by site of recruitment.

Clinical Diagnosis		Adjudicated diagnosis	Congo Red test result		Case notes
***Bangladesh***					
CONTROL *N* = 98		NO PE *n* = 81	**NO, *n*** **=** **70** **True negative** YES, *n* = 11 False positive		**Unanimous concordance ruling out PE.** Cases in this category underwent medically indicated delivery due to prior c/s. All tested weak positive.
		Other co-morbid conditions (*n* = 17)	NO, *n* = 9 True negative YES, *n* = 8 False positive		CRD concurred with adjudicated diagnosis. Cases included severe oligohydramnios (*n* = 1), case with history of hypertension and heart disease (*n* = 1), gestHT without a diagnosis of PE, medically indicated delivery for reduced fetal movement (*n* = 1). All 8 cases tested weak positive.
CASE *N* = 98		NO PE *n* = 8	NO=1 True negative YES=7 False positive		CRD concurred with adjudicated diagnosis of chronic hypertension while managing team's call was preeclampsia, uncategorized. Case adjudications was gestational hypertension (*n* = 1) while managing team's diagnosis was severe preeclampsia. Case adjudication was atypical PE or PE mimics (*n* = 2) while managing team's diagnosis was eclampsia. Case adjudication was idiopathic isolated proteinuria (*n* = 4) while the managing team's diagnosis was sPE, HELLP and PE uncategorized. All 7 cases tested weak positive.
		YES PE *n* = 90	NO, *n* = 14 False negative **YES, *n*** **=** **76** **True positive**		Case adjudication was sPE no PLTC (*n* = 5) and sPE with PLTC (*n* = 9) including a maternal death due to cardiac arrest. 5 had history of eclampsia prior to admission. 6 underwent MIDPE. 7 with a GA <34 weeks. **Unanimous concordance ruling in PE.**
***Mexico***					
CONTROL *N* = 107	NO PE *n* = 92		**NO, *n*** **=** **69** **True negative** YES, *n* = 23 False positive	**Unanimous concordance ruling out PE.** Included cases with medically indicated delivery for failure to progress (*n* = 3), PROM (*n* = 1), CPD (*n* = 1) and short inter-pregnancy interval (*n* = 1). One case experienced a pph and one case with diabetes.	
	Other co-morbid conditions (*n* = 11)		NO, *n* = 10 True negative YES, *n* = 1 False positive	CR test concurred with adjudicated diagnosis. Adjudicated diagnoses were hypertension (*n* = 4), oligohydramnios (*n* = 5) or abruption (*n* = 1). Case with weak positive test underwent medically indicated delivery for oligohydramonius.	
	Unclassifiable (unknown BP) (*n* = 4)		NO, *n* = 3 YES, *n* = 1	Unknown BP at time of delivery and inadequate clinical data to rule out other co-morbid conditions or PE.	
CASE *N* = 106	NO PE *n* = 41		NO, *n* = 28 True negative YES, *n* = 13 False positive	CR test concurred with adjudicated diagnosis. Adjudicated diagnosis was of gestHTN (*n* = 17), amPE without PLTC (*n* = 4), IDP (*n* = 4) or no PE criteria (*n* = 3) while managing team's call was of mPE, SPE or uncategorized PE. 17 cases underwent MIDPE. Adjudicated diagnosis included gestHTN (*n* = 5), amPE with PLTC (*n* = 1), amPE without pLTC (*n* = 1), IDP (*n* = 5) and no PE criteria (*n* = 1). The three cases with a strong positive result (E) had an adjudicated diagnosis of idiopathic proteinuria.	
	Yes PE *n* = 65		NO, *n* = 27 False negative **YES, *n*** **=** **38** **True positive**	Cases included mPE (*n* = 8), SPE without PLTC (*n* = 13), SPE with PLTC (*n* = 3), spPE (*n* = 3). **Unanimous concordance ruling in PE**	

*Positive test defined as score C-F. SPE = severe preeclampsia, PLTC=preeclampsia-related life threatening conditions, gestHTN=gestational hypertension, spPE=superimposed preeclampsia, amPE=atypical preeclampsia or preeclampsia mimic, MIDPE=medically indicated delivery for preeclampsia, PROM=premature rupture of membranes; CPD= Cephalopelvic disproportion.

Table 4 presents the attending team's diagnosis, final adjudicated diagnosis and result of the GV-005 test for the Mexico cohort. In Mexico, 92 controls (86%) were adjudicated as non-preeclampsia and of these 69 (75%) tested negative for congophilia. Case by case analysis found that adjudicated control patients with a positive test result included cases with a medically-indicated delivery for PROM, macrosomia and one case with diabetes. Among controls with other co-morbid conditions (*n* = 11), the GV-005 concurred with the adjudicated diagnosis of non-preeclampsia and included patients with hypertension, oligohydramnios and abruption. Four controls in the Mexico cohort were lacking data on the blood pressure at the time of delivery and inadequate clinical data to rule out other co-morbid conditions or preeclampsia. Amongst the pre-eclampsia cases recruited in Mexico, 41 (39%) were adjudicated as non-preeclampsia. In this sub-group, 21 (68%) tested negative for congophilia. Most of these cases had an adjudicated diagnosis of gestational hypertension, atypical preeclampsia or preeclampsia mimics or idiopathic proteinuria. Approximately half of which (41%) underwent a medically-indicated delivery for preeclampsia. Among the Mexico cases with an adjudicated diagnosis of preeclampsia (*n* = 65), 58% tested positive for congophilia while 27 (42%) had a negative GV-005 test result. This sub-group included cases of preeclampsia with and without severe features and with and without PLTC. In both the case and control groups, most false positive and false negative results occurred in the context of gestational hypertension, chronic hypertension or other preeclampsia mimics.

## Discussion

This is the first study using a beta prototype of a point-of-care diagnostic device for detection of urine congophilia in a low resource setting that could potentially be manufactured at scale for commercial purposes. We also report large discrepancies in the preeclampsia diagnostic criteria between the study sites and when compared to the standard ACOG criteria.

Preeclampsia is classically defined as new onset hypertension and proteinuria after 20 weeks of gestation [20]. International professional bodies have sought to refine their diagnostic criteria in recent years [21, 22, 23, 24]. However, as our study has shown, the practice of diagnosis may vary widely depending on local clinical practice and guidelines and, in application, may result in misdiagnosis. A lack of detailed laboratory workups in both of our study settings meant that diagnoses were based often on clinical signs and symptoms resulting in some misclassification. Given this variability in diagnostic practice, we used an adjudicated diagnosis to serve as a standard reference for cases in both settings. We found more discordance between the clinical diagnosis of the treating team and the adjudicated diagnosis among preeclampsia cases recruited in Mexico compared to Bangladesh. In the Mexico sample, 39% of cases were reclassified compared to only 8% of cases in the Bangladesh (*p* = 0.0001). Bangladesh cases were more severe and thus less likely to be clinically misclassified at enrollment. In over two-thirds of the cases misclassified, the CR test agreed with the adjudicated diagnosis. Based on these findings, we believe the CR test could be a useful tool for triaging patients in the context of a care setting such as Mexico where many patients present with existing hypertension and ruling out preeclampsia is challenging. This finding echoes results from a prior study with the paper-based CR test in a US hospital [11]. However, the GV-005 device used here had a higher false-positive rate at both centers compared with prototype tested in the USA. Possible explanations include: the device was not calibrated to the patient population; imperfect reading especially in Mexico where there was a higher turnover of staff performing the test; labor or pre-labor might induce a low degree of congophilia; and women with oligohydramnios were under-diagnosed for preeclampsia

The study was conducted in two settings with very different patient populations. Preeclampsia cases in the Bangladesh cohort presented at a more advanced stage of disease compared to Mexico and cases in Mexico were more likely to be diagnosed as preeclampsia on the basis of elevated blood pressure alone. Over half of Bangladeshi cases had preeclampsia related life-threatening conditions compared to only 11% of adjudicated cases in Mexico. The specificity and sensitivity of the test can be affected by the patient characteristics in different settings because each setting has a different mix of patients [25]. Severe cases, as in Bangladesh, are easier to detect, resulting in an increased sensitivity, while healthy controls could reduce the number of false positives resulting in an overestimate the specificity of the test. While cases in both sites were significantly more likely to have a positive test result, the odds of a positive test among cases vs controls in Bangladesh was much higher than in Mexico (Bangladesh: 323 vs Mexico: 20). The results may not be representative or applicable in other populations. As observed in our study the patient population affects the performance of the test particularly in low-resourced settings when clinical data to rule out or to rule in preeclampsia based on other laboratory tests (such as 24 h proteinuria and repeated blood work) is missing. Although these data do provide information on the suitability of the test in different sub-groups of patients, a cohort study could thus provide a better estimate of test performance [26].

Laboratory technicians performing the test were trained and provided with a visual chromatic scale to guide scoring of tests. However, there was room for reader error in test interpretation and distinguishing between readings of a B vs C or C vs. D. As a result, there was the possibility of misclassification of a positive vs. a negative test. The user feedback provided during this project was applied by GestVision (USA) to develop a third generation device, the GV-010, which is intended for use at the bedside and standardized commercial manufacturing. The new test has a dichotomous (positive/negative) result and a hue guide which the operator can place by the reaction window to aid in visual interpretation of the observed pattern.

The study was powered on a combined sample size including cases and controls from both study sites. However, variation in the implementation of the study and the profile of the patient population in the two sites required us to stratify the analysis. Where possible, patients were recruited with a single case and a control recruited as a pair. In Bangladesh research staff effectively recruited cases consecutively. However, changes in staffing at the Mexico site and sampling of urine postpartum resulted in gaps and an extended recruitment period further contributing to some variation in the case definition. Our adjudicated case definition helped to address some of these inter-site differences, however, it also resulted in a reduction in the number of eligible cases in Mexico. The stratification reduced the study power resulting in wide confidence intervals in the OR at both sites, which could reduce the accuracy of the estimates.

In sum, our study found that preeclampsia is characterized by urine congophilia which can be rapidly identified using a beta prototype of a lateral flow diagnostic device, GV-005. This embodiment of the CR test could be a useful tool to aid in the diagnosis of patients with preeclampsia and identification of patients that required additional care. Delivery triage may not be very useful. Future research should explore the feasibility and acceptability of the device in settings at lower levels of the health care system in order to identify patients earlier during the antenatal period and help to ensure that patients receive appropriate care.

## Data sharing statement

Data are available upon reasonable request. A member of the study team must be involved in the analysis of the data.

## Contributions

IB and CB had the original idea for the study. IB, CB, TE, HB, MB, AR, JP, and BW formed the trial management team with input from other co-investigators as required. HB and MB were the study monitors. IB and CB performed the case adjudication. HB and IB conducted the main analysis and HB wrote the first draft of the clinical paper. All authors reviewed and accepted the paper prior to submission.

## Declaration of Competing Interest

Dr. Irina Buhimschi reports grants from Saving Lives at Birth (SLaB) partners including USAID, Bill & Melinda Gates Foundation, Goverments of Canada, Norway and UK DFID, during the conduct of the study; other from GestVision Inc. In addition, Dr. Irina Buhimschi has a patent US Patent Number 8263,342 with royalties paid by GestVision Inc, a patent US Patent Number 9229,009 with royalties paid by GestVision Inc and by Shuwen Biotech, a patent U.S. Patent Number 10,324,094 with royalties paid by GestVision Inc, and a patent European patent No 3,129,779 with royalties paid by GestVision Inc. These royalties are paid to Yale University who disburses a percentage to inventors and co-inventors. The Congo Red devices used in this study were purchased from GestVision Inc who had no input in data interpretation or decision to submit for publication.

Dr. Catalin Buhimschi reports grants from Saving Lives at Birth (SLaB) partners including USAID, Bill & Melinda Gates Foundation, Goverments of Canada, Norway and UK DFID, during the conduct of the study; other from GestVision Inc. In addition, Dr. Catalin Buhimschi has a patent US Patent Number 8263,342 with royalties paid by GestVision Inc, a patent US Patent Number 9229,009 with royalties paid by GestVision Inc and by Shuwen Biotech, a patent U.S. Patent Number 10,324,094 with royalties paid by GestVision Inc, and a patent European patent No 3,129,779 with royalties paid by GestVision Inc. These royalties are paid to Yale University who disburses a percentage to inventors and co-inventors. The Congo Red devices used in this study were purchased from GestVision Inc who had no input in data interpretation or decision to submit for publication.

Dr. Easterling reports personal fees from DiabetOmics, Inc., outside the submitted work.

The other authors have no conflicts to declare.

## Appendix Supplementary materials

Image, application 1

Image, application 2

Image, application 3

Image, application 4

## References

[1] Souza J.P., Gülmezoglu A.M., Vogel J. (2013). Moving beyond essential interventions for reduction of maternal mortality (the WHO Multicountry Survey on Maternal and Newborn Health): a cross-sectional study. Lancet.

[2] Chan P., Brown M., Simpson J.M., Davis G. (2005). Proteinuria in pre-eclampsia: how much matters?. BJOG.

[3] Murray N., Homer C.S., Davis G.K., Curtis J., Mangos G., Brown M.A. (2002). The clinical utility of routine urinalysis in pregnancy: a prospective study. Med J Aust.

[4] Kamińska J., Dymicka-Piekarska V., Tomaszewska J. et al. Diagnostic utility of protein to creatinine ratio (P/C ratio) in spot urine sample within routine clinical practice, Crit Rev Clin Lab Sci, 57:5, 345–64, DOI: 10.1080/10408363.2020.1723487 10.1080/10408363.2020.1723487 32058809

[5] Sibai B.M., Stella C.L. (2009). Diagnosis and management of atypical preeclampsia-eclampsia. Am J Obstet Gynecol.

[6] Thaddeus S., Maine D.. Too far to walk: maternal mortality in context. Soc Sci Med 1994; 38: 1091–110. 10.1016/0277-9536(94)90226-7 8042057

[7] Majors C.E., Smith C.A., Natoli M.E., Kundrod K.A., Richards-Kortum R. (2017). Point-of-care diagnostics to improve maternal and neonatal health in low-resource settings. Lab Chip.

[8] Buhimschi I.A., Zhao G., Funai E.F. (2008). Proteomic profiling of urine identifies specific fragments of SERPINA1 and albumin as biomarkers of preeclampsia. Am J Obstet Gynecol.

[9] Buhimschi I.A., Nayeri U.A., Zhao G. (2014). Protein misfolding, congophilia, oligomerization, and defective amyloid processing in preeclampsia. Sci Transl Med.

[10] Buhimschi I.A., Buhimschi C.S., Tagare H., Choma M., Jonas S.. Methods and compositions for detecting misfolded proteins. U.S. Patent Number 10,324,094 Filed April 10, 2014, issued June 18, 2019.

[11] Rood K.M., Buhimschi C.S., Dible T., Webster S., Zhao G., Samuels P., Buhimschi I.A. (2019). Congo red dot paper test for antenatal triage and rapid identification of preeclampsia. E Clin Med.

[12] Sammar M., Syngelaki A., Sharabi-Nov A., Nicolaides K., Meiri H. (2017). Can staining of damaged proteins in urine effectively predict preeclampsia?. Fetal Diagn Ther.

[13] Nagarajappa C., Rangappa S.S., Suryanarayana R., Balakrishna S. (2018). Urinary congophilia in preeclampsia: experience from a rural tertiary-care hospital in India. Pregnancy Hypertens.

[14] McCarthy F.P., Adetoba A., Gill C. (2016). Urinary congophilia in women with hypertensive disorders of pregnancy and preexisting proteinuria or hypertension. Am J Obstet Gynecol.

[15] Li X.-.M., Liu X.-.M., Xu J., Du J., Cuckle H. (2020). Late pregnancy screening for preeclampsia with a urinary point-of-care test for misfolded proteins. PLoS ONE.

[16] Davis, W.L., Levenson D. Device for detecting misfolded proteins and methods of use. U.S. Patent Number 10,564,153 Filed 2015, issued May 7, 2020.

[17] Fryer H.J.L., Rood K.M., Davis W.L., Buhimschi C.S., Buhimschi I.A. (2019). Evaluation of a rapid urine test for preeclampsia in an antenatal triage population.

[18] Kahan B.C., Feagan B., Jairath V. (2017). A comparison of approaches for adjudicating outcomes in clinical trials. Trials.

[19] American College of Obstetricians and Gynecologists (2013). Task force on hypertension in pregnancy. Hypertension in pregnancy. Report of the American college of obstetricians and gynecologists task force on hypertension in pregnancy. Obstet Gynecol.

[20] National High Blood Pressure Education Program Working Group on High Blood Pressure in Pregnancy (2000). Report of the national high blood pressure education program working group on high blood pressure in pregnancy. Am J Obstet Gynecol.

[21] Brown M.A., Magee L.A., Kenny L.C., Karumanchi S.A., McCarthy F.P., Saito S., Hall D.R., Warren C.E., Adoyi G., Ishaku S. (2018 Jul). International society for the study of hypertension in pregnancy (ISSHP). The hypertensive disorders of pregnancy: ISSHP classification, diagnosis & management recommendations for international practice. Pregnancy Hypertens.

[22] Webster K., Fishburn S., Maresh M., Findlay S.C., Chappell L.C., Guideline Committee (2019 Sep 9). Diagnosis and management of hypertension in pregnancy: summary of updated NICE guidance. BMJ.

[23] American College of Obstetricians and Gynecologists' Committee on Practice Bulletins—Obstetrics (2020 Jun). Gestational hypertension and preeclampsia: ACOG practice bulletin, number 222. Obstet Gynecol.

[24] Poon L.C., Shennan A., Hyett J.A., Kapur A., Hadar E., Divakar H., McAuliffe F., da Silva Costa F., von Dadelszen P., McIntyre H.D., Kihara A.B., Di Renzo G.C., Romero R., D'Alton M., Berghella V., Nicolaides K.H., Hod M. (2019). The international federation of gynecology and obstetrics (FIGO) initiative on pre-eclampsia: a pragmatic guide for first-trimester screening and prevention. Int J Gynaecol Obstet.

[25] Li J., Fine J.P. (2011). Assessing the dependence of sensitivity and specificity on prevalence in metaanalysis. Biostatistics.

[26] Rutjes A.W., Reitsma J.B., Vandenbroucke J.P., Glas A.S., Bossuyt P.M. (2005). Case-control and two-gate designs in diagnostic accuracy studies. Clin Chem.

