# Supplementary material for: Congo red test for identification of preeclampsia: Results of a prospective diagnostic case-control study in Bangladesh and Mexico
Source: eClinicalMedicine. 2020 Dec 22;31:100678. doi: 10.1016/j.eclinm.2020.100678 (PMC7770484; doi:10.1016/j.eclinm.2020.100678)
Supplement: Supplementary file 1 [file mmc1.pptx]

## Slide 1
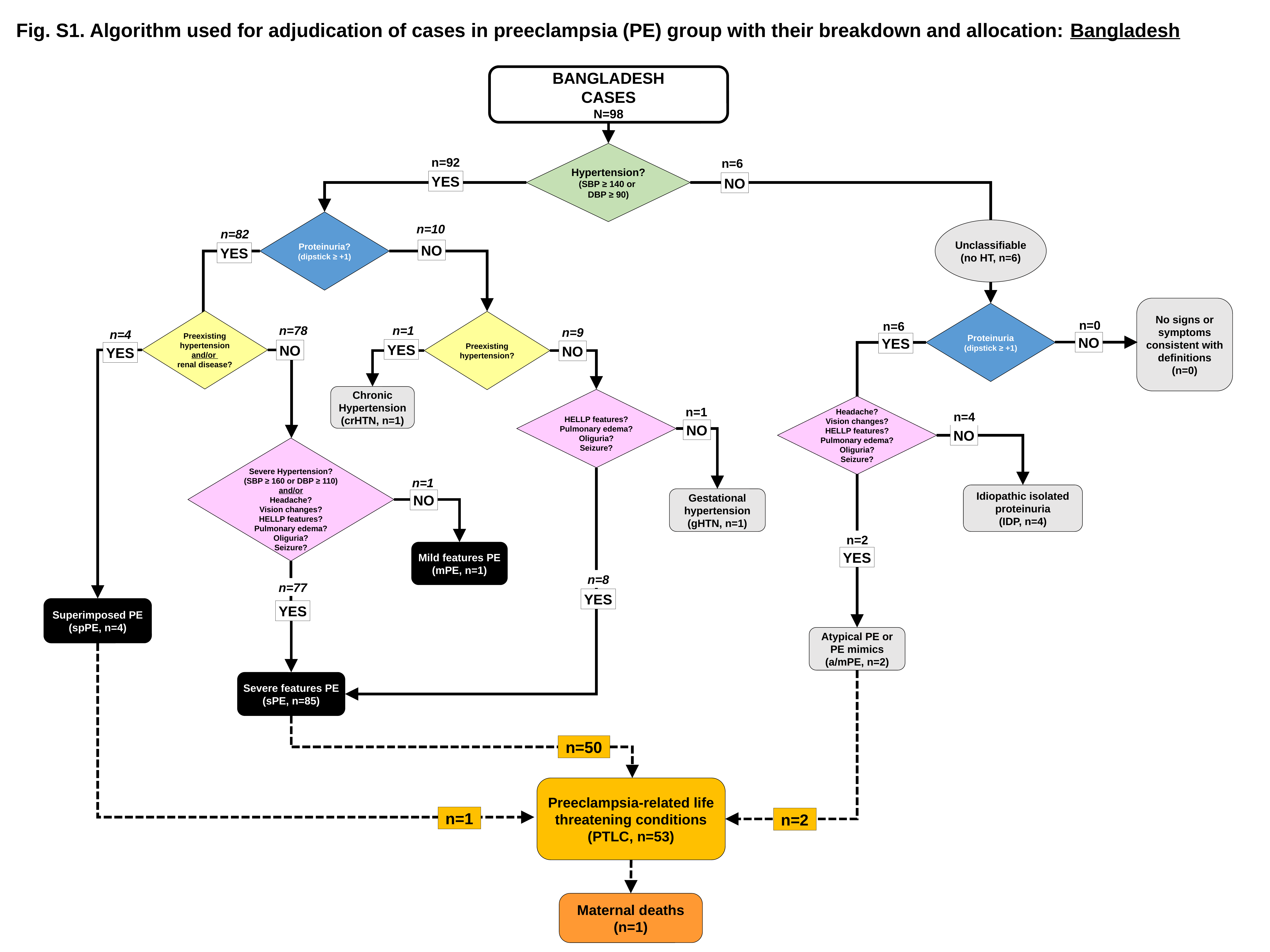

Fig. S1. Algorithm used for adjudication of cases in preeclampsia (PE) group with their breakdown and allocation: Bangladesh
BANGLADESH
CASES
N=98
Hypertension?
(SBP ≥ 140 or
DBP ≥ 90)
n=92
n=6
YES
NO
Proteinuria?
(dipstick ≥ +1)
n=10
Unclassifiable (no HT, n=6)
n=82
NO
YES
No signs or symptoms consistent with definitions
(n=0)
Proteinuria
(dipstick ≥ +1)
Preexisting hypertension and/or
renal disease?
Preexisting hypertension?
n=0
n=6
n=78
n=1
n=9
n=4
NO
YES
YES
NO
NO
YES
Chronic
Hypertension
(crHTN, n=1)
HELLP features?
Pulmonary edema?
Oliguria?
Seizure?
Headache?
Vision changes?
HELLP features?
Pulmonary edema?
Oliguria?
Seizure?
n=1
n=4
NO
NO
Severe Hypertension? (SBP ≥ 160 or DBP ≥ 110)
and/or
Headache?
Vision changes?
HELLP features?
Pulmonary edema?
Oliguria?
Seizure?
n=1
Idiopathic isolated
proteinuria
(IDP, n=4)
Gestational hypertension
(gHTN, n=1)
NO
n=2
Mild features PE (mPE, n=1)
YES
n=8
n=77
YES
Superimposed PE
(spPE, n=4)
YES
Atypical PE or PE mimics
(a/mPE, n=2)
Severe features PE
(sPE, n=85)
 n=50
Preeclampsia-related life threatening conditions (PTLC, n=53)
 n=1
 n=2
Maternal deaths
(n=1)
