# Supplementary material for: Congo red test for identification of preeclampsia: Results of a prospective diagnostic case-control study in Bangladesh and Mexico
Source: eClinicalMedicine. 2020 Dec 22;31:100678. doi: 10.1016/j.eclinm.2020.100678 (PMC7770484; doi:10.1016/j.eclinm.2020.100678)
Supplement: Supplementary file 2 [file mmc2.pptx]

## Slide 1
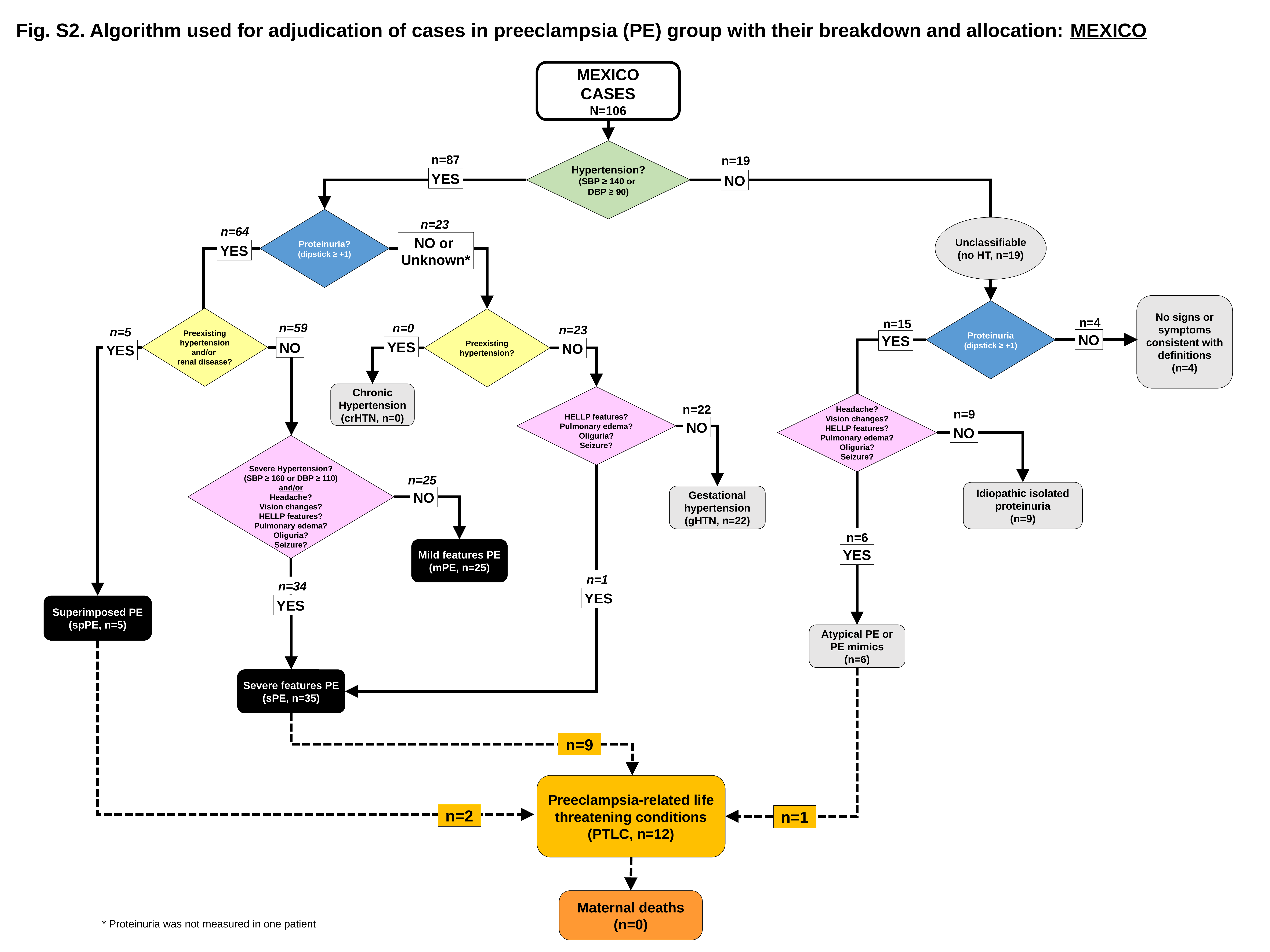

Fig. S2. Algorithm used for adjudication of cases in preeclampsia (PE) group with their breakdown and allocation: MEXICO
MEXICO
CASES
N=106
Hypertension?
(SBP ≥ 140 or
DBP ≥ 90)
n=87
n=19
YES
NO
Proteinuria?
(dipstick ≥ +1)
n=23
Unclassifiable (no HT, n=19)
n=64
NO or
Unknown*
YES
No signs or symptoms consistent with definitions
(n=4)
Proteinuria
(dipstick ≥ +1)
Preexisting hypertension and/or
renal disease?
Preexisting hypertension?
n=4
n=15
n=59
n=0
n=23
n=5
NO
YES
YES
NO
NO
YES
Chronic
Hypertension
(crHTN, n=0)
HELLP features?
Pulmonary edema?
Oliguria?
Seizure?
Headache?
Vision changes?
HELLP features?
Pulmonary edema?
Oliguria?
Seizure?
n=22
n=9
NO
NO
Severe Hypertension? (SBP ≥ 160 or DBP ≥ 110)
and/or
Headache?
Vision changes?
HELLP features?
Pulmonary edema?
Oliguria?
Seizure?
n=25
Idiopathic isolated
proteinuria
(n=9)
Gestational hypertension
(gHTN, n=22)
NO
n=6
Mild features PE (mPE, n=25)
YES
n=1
n=34
YES
YES
Superimposed PE
(spPE, n=5)
Atypical PE or PE mimics
(n=6)
Severe features PE
(sPE, n=35)
 n=9
Preeclampsia-related life threatening conditions (PTLC, n=12)
 n=2
 n=1
Maternal deaths
(n=0)
* Proteinuria was not measured in one patient
