# Supplementary material for: Congo red test for identification of preeclampsia: Results of a prospective diagnostic case-control study in Bangladesh and Mexico
Source: eClinicalMedicine. 2020 Dec 22;31:100678. doi: 10.1016/j.eclinm.2020.100678 (PMC7770484; doi:10.1016/j.eclinm.2020.100678)
Supplement: Supplementary file 3 [file mmc3.pdf]

Supplement A. Technical cross-validation of the GV-005 test.

Urine samples were collected prospectively from 71 women who were evaluated for preeclampsia at The Ohio State Wexner Medical Center. These cases were different from the ones included in the previously published study[1] but the protocol methodological protocol was similar except for the use of GV-005 in research laboratory setting.

Fresh urine was applied concomitantly to the GV-005 and the CRD Paper Test.[1] Results were scored independently, at 3 min. using a chromatic **6-level visual analytic scale** (**Fig. 3**). The CRD nitro-cellulose array was run in the research laboratory and the test result (% CRR) was calculated using the automated protocol cell phone app as previously published.[2] A %CRR >15 was considered positive. Correlation among test results, agreement of positive and negative scores along with user experience was recorded. The nitro-cellulose %CRR values for the specimens ranged from 0-89% with 40/71 measuring as positive for urine congophilia (%CRR>15%). There was a significant difference in congophilia level between positive (%CRR: 60.4±3.0) and negative (%CRR: 4.8±0.7) specimens. The GestAssured™ device score correlated significantly with %CRR (0.865, P<0.001) and the paper-based CRD Simple Test Score (r=0.799, P<0.001) with "excellent-level" agreement (kappa=0.86). Users reported a “high” degree of satisfaction with the device’s design, and performance. The conclusion was that the **GV-005** device appears to be a reproducible, qualitative, LFA diagnostic aid that detects congophilia in urine of women with PE, similarly to paper-based CRD and laboratory nitro-cellulose %CRR.

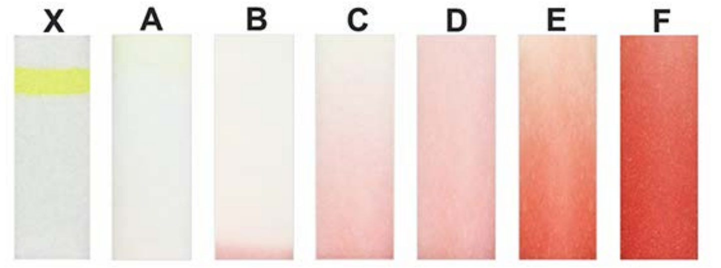

Fig. 3. The GV-005 test result is interpreted visually as 5 classes (A-F) against a visual analogue scale. The test marked as X shows the unreacted test with noticeable yellow band.

Development of the 6-level visual analytic scale for read-out of the GV-005 result and of the materials necessary to perform the test and data collection at international sites.

Although this is still to be demonstrated, the reaction pattern of urine+Congo Red on a lateral flow chromatographic surface is the result of many parameters of which chemical qualities of the surface, concentration of aggregates of misfolded proteins, their size and their chemical characteristics are anticipated to play defined roles. Because the volume of urine applied to the GV-005 test the volume of urine (~70 µL) is larger than in the CRD Paper Test and the migration distance is longer, a wider range of result patterns was observed during the above cross-validation study. These patterns correlated with disease severity and appeared to carry additional information comparing to the 3 types of result scores CRD Paper Test (negative, weak-positive, strong-positive). Due to the exploratory nature of the study in LMIC settings, the agreement among team members was to record the various categories as close as possible to the visual image of the reacted test and let the clinical trials inform on the significance (or lack thereof) of each pattern. Instead of a ordinal scale (0-5) the visual chromatic scale had 6 categories marked A to F as we are not yet sure that from B, C to D there is an incremental and direct correlation with disease severity.

[1] Kara M. Rood, Catalin S. Buhimschi, Theresa Dible, Shaylyn Webster, Guomao Zhao, Philip Samuels, Irina A. Buhimschi. Congo red dot paper test for antenatal triage and rapid identification of preeclampsia. E Clin Med. 2019 Feb;8:47-56. DOI: 10.1016/j.eclinm.2019.02.004

[2] Jonas SM, Deserno TM, Buhimschi CS, Makin J, Choma MA, Buhimschi IA. Smartphone-based diagnostic for preeclampsia: an mHealth solution for administering the Congo Red Dot (CRD) test in settings with limited resources. J Am Med Inform Assoc. 2016 Jan;23(1):166-73. doi: 10.1093/jamia/ocv015. Epub 2015 May 29. PMID: 26026158.
