# Supplementary material for: Congo red test for identification of preeclampsia: Results of a prospective diagnostic case-control study in Bangladesh and Mexico
Source: eClinicalMedicine. 2020 Dec 22;31:100678. doi: 10.1016/j.eclinm.2020.100678 (PMC7770484; doi:10.1016/j.eclinm.2020.100678)
Supplement: Supplementary file 4 [file mmc4.docx]

**Table S1. Background characteristics and delivery outcomes for preeclampsia cases by site of enrollment**

| **Variable** | **Mexico (n=106)** | | **Bangladesh (n=98)** | | **p-value** |
| --- | --- | --- | --- | --- | --- |
|  | **n** | **%** | **n** | **%** |  |
| Gestational age (completed weeks) |  |  |  |  | <0.001 |
| <34 weeks | 14 | 13.2 | 50 | 51 |  |
| 34-36 weeks | 18 | 17 | 32 | 32.7 |  |
| 37-38 weeks | 33 | 31.1 | 6 | 6.1 |  |
| 39> weeks | 41 | 38.7 | 10 | 10.2 |  |
| Signs and symptoms at time of diagnosis |  |  |  |  |  |
| Elevated BP | 88 | 83 | 96 | 98 | <0.001 |
| Proteinuria | 6 | 5.7 | 94 | 95.9 | <0.001 |
| Headache | 23 | 21.7 | 74 | 75.5 | <0.001 |
| Changes in vision | 2 | 1.9 | 65 | 66.3 | <0.001 |
| Abdominal pain | 11 | 10.4 | 1 | 1 | 0.005 |
| Nausea/vomiting | 2 | 1.9 | 29 | 29.6 | <0.001 |
| Seizure | 1 | 0.9 | 39 | 39.8 | <0.001 |
| Systolic BP (mmHg) at time of diagnosis |  |  |  |  | <0.001 |
| <139 | 26 | 24.5 | 8 | 8.2 |  |
| 140-159 | 51 | 48.1 | 33 | 33.7 |  |
| >160 | 29 | 27.4 | 57 | 58.2 |  |
| Diastolic BP (mmHg) at time of diagnosis |  |  |  |  | <0.001 |
| <89 | 32 | 30.2 | 10 | 10.2 |  |
| 90-109 | 62 | 58.5 | 36 | 36.7 |  |
| >110 | 12 | 11.3 | 52 | 53.1 |  |
| Proteinuria at time of diagnosis |  |  |  |  |  |
| Nil or Trace | 26 | 24.5 | 10 | 10.2 | <0.001 |
| 1 | 43 | 40.6 | 10 | 10.2 |  |
| 2 | 14 | 13.2 | 8 | 8.2 |  |
| >+3 | 22 | 20.9 | 70 | 70.4 |  |
| Woman received MgSO4 in 6h preceding enrollment (n,%) | 62 | 58.5 | 90 | 91.8 | <0.001 |
| Woman received antihypertensive drug in 6h hours preceding enrollment | 87 | 82.1 | 98 | 100 | <0.001 |
| Mode of delivery |  |  |  |  | <0.001 |
| Vaginal delivery | 23 | 21.7 | 48 | 49 |  |
| Forceps or vacuum delivery | 2 | 1.9 | 0 | 0 |  |
| C-section | 81 | 76.4 | 50 | 51 |  |
| Experienced preeclampsia-related life threatening complication | 53 | 54 | 12 | 11 | <0.001 |
| Admission to ICU up to discharge | 32 | 30.2 | 2 | 2 | <0.001 |
